# Molecular characterization and phylogenetic diversity of fowl aviadenovirus serotype 8b associated with inclusion body hepatitis in Thai chickens

**DOI:** 10.14202/vetworld.2025.1685-1693

**Published:** 2025-06-26

**Authors:** Tawatchai Pohuang, Kanlaya Worawong, Kingkarn Sarachu, Duangdaow Khunbutsri, Sucheeva Junnu

**Affiliations:** 1 Division of Livestock Medicine, Faculty of Veterinary Medicine, Khon Kaen University, Khon Kaen 40002, Thailand; 2 Laboratory and Laboratory Animals, Faculty of Veterinary Medicine, Khon Kaen University, Khon Kaen 40002, Thailand; 3 Veterinary Diagnostic Laboratory, Faculty of Veterinary Medicine, Khon Kaen University, Khon Kaen 40002, Thailand

**Keywords:** chicken, fowl aviadenovirus, hexon gene, inclusion body hepatitis, phylogenetic analysis, Thailand

## Abstract

**Background and Aim:**

Inclusion body hepatitis (IBH) is an acute and economically significant disease in poultry, caused by fowl aviadenovirus (FAdV), particularly serotypes belonging to species D and E. In Thailand, outbreaks of IBH associated with FAdV have been sporadically reported since 2007, yet comprehensive molecular surveillance remains limited. This study aimed to detect, molecularly characterize, and phylogenetically analyze FAdV strains associated with IBH in commercial broiler and breeder chicken farms across four provinces in Thailand.

**Materials and Methods:**

A total of 28 liver samples were collected from chickens exhibiting clinical signs of IBH in Kanchanaburi, Chonburi, Lopburi, and Songkhla Provinces between June and December 2024. Gross and histopathological examinations were conducted, followed by a polymerase chain reaction targeting the hexon gene. Six representative positive samples were subjected to DNA sequencing and phylogenetic analysis using MEGA 11 software. Comparative amino acid sequence analysis was also performed to evaluate potential strain divergence.

**Results:**

All 28 samples tested positive for FAdV, with gross pathology revealing pale, friable, and hemorrhagic livers. Histopathological analysis confirmed multifocal hepatic necrosis with characteristic basophilic intranuclear inclusion bodies. Sequencing and phylogenetic analysis identified all isolates as FAdV species E, serotype 8b. The isolates shared 94.73%–100% nucleotide similarity with reference strains from China, Indonesia, and Turkey. Phylogenetic clustering revealed two distinct groups among the Thai isolates, associated with specific amino acid substitutions at positions 17, 19, 20, 22, and 37 of the hexon gene.

**Conclusion:**

This study represents the first report of FAdV-E serotype 8b as the causative agent of IBH outbreaks in multiple commercial broiler and breeder chicken farms in Thailand. The detection of two phylogenetically distinct groups suggests the concurrent circulation of genetically diverse strains, potentially linked to vertical transmission routes. These findings underscore the urgent need for molecular surveillance, vaccination strategies utilizing local strains, and enhanced biosecurity measures to mitigate the spread of FAdV in the Thai poultry industry.

## INTRODUCTION

Inclusion body hepatitis (IBH) is an acute viral disease in chickens caused by fowl aviadenovirus (FAdV) [1]. FAdVs belong to the family *Adenoviridae* and are non-enveloped, icosahedral viruses possessing a double-stranded DNA genome. Their capsids are composed of 720 hexons organized into 240 trimers and 12 vertex pentons [2]. The primary structural proteins of the FAdV capsid include hexons, fibers, and the penton base. Based on genomic sequencing, FAdVs are classified into five species (A–E), encompassing 12 serotypes (FAdV-1–8a and 8b–11) as determined through cross-neutralization assays [3].

First identified in the United States during the 1960s [4], IBH has since become a globally distributed disease, leading to substantial economic losses in countries such as Canada [5], Japan [6], China [7], South Korea [8], Indonesia [9], Iraq [10], Poland [11], Greece [12], Bangladesh [13], and India [14, 15].

Clinically, IBH is associated with poor-quality 1-day-old chicks and flock mortality rates ranging from 5% to 10% within the first 3 weeks of life, occasionally escalating to as high as 30% [16]. In broiler breeder flocks, the disease is marked by reduced egg production, diminished hatchability, and delayed hatching [12]. Gross pathological findings often include hepatomegaly with pale, friable livers exhibiting subcapsular petechial hemorrhages [17]. Microscopically, the presence of basophilic intranuclear inclusion bodies within hepat-ocytes serves as a pathognomonic feature of IBH [12].

Diagnosis of IBH can be achieved through various approaches, including molecular and serological methods [18]. Polymerase chain reaction (PCR) targeting the hexon gene is commonly employed for specific detection of FAdV [19]. In addition, sequencing of the hypervariable region in the loop-1 domain of the hexon gene enables differentiation among FAdV species (A–E) and serotypes [20, 21]. Serological assays, such as the serum neutralization test, are also utilized to determine FAdV serotypes [22].

The predominant FAdV serotypes implicated in IBH include FAdV-D (serotypes 2, 3, 9, and 11) and FAdV-E (serotypes 6, 7, 8a, and 8b) [23]. In Thailand, the first confirmed outbreak of IBH attributed to FAdV serotype 2 occurred in 2007 among commercial broiler breeder farms, causing a severe economic impact [24, 25]. Affected flocks demonstrated elevated mortality in 5–7-day-old chicks, poor chick quality, and delayed hatching.

Despite the growing recognition of FAdV as a major etiological agent of IBH in poultry worldwide, there remains a significant paucity of comprehensive molecular and phylogenetic data on circulating FAdV strains in Thailand. Previous reports from the country have predominantly focused on isolated outbreaks, with limited emphasis on genotypic characterization and serotype distribution. Notably, although serotype 2 of FAdV-D was identified during the first documented outbreak in Thai broiler breeder flocks in 2007, recent evidence suggests the emergence and potential co-circulation of other virulent serotypes, including FAdV-8b of species E. Furthermore, while international studies have extensively explored the genetic variability and pathogenic potential of FAdV-8b strains from regions such as Indonesia, China, and Turkey, the phylogenetic positioning and molecular features of Thai isolates remain underexplored. The absence of such epidemiological and genetic data impedes the development of region-specific diagnostic tools and effective vaccination strategies. In addition, the role of vertical transmission in the spread of genetically distinct FAdV-8b variants across geographically dispersed poultry farms in Thailand has not been adequately investigated.

The present study was conducted to address this knowledge gap by investigating outbreaks of IBH in commercial broiler and breeder flocks across multiple provinces in Thailand. Specifically, the study aimed to detect and identify the FAdV strains present in affected chickens through histopathological and molecular analyses. PCR targeting the hexon gene was employed for preliminary identification, followed by sequencing and phylogenetic characterization of representative isolates. A key objective was to determine the genetic diversity and phylogenetic relationships of the FAdV strains, with a particular focus on serotype 8b, and to evaluate potential amino acid variations within the hexon gene that may be indicative of strain divergence. By elucidating the molecular characteristics and trans-mission dynamics of circulating FAdV serotypes, the study aims to enhance understanding of the epide-miology of IBH in Thailand and to inform future disease surveillance, control, and vaccine development efforts.

## MATERIALS AND METHODS

### Ethical approval

This study was conducted in accordance with the ethical standards for animal research. The research protocol was approved by the Institutional Animal Care and Use Committee of Khon Kaen University under record number IACUC-KKU (C)-55/67.

### Study period and sampling locations

From June to December 2024, a total of 28 chicken carcasses were collected from 11 farms comprising six commercial broiler and five broiler breeder operations. These farms were located across four provinces in Thailand: Kanchanaburi (KCB), Chonburi (CB), Lopburi (LB), and Songkhla (SK), representing the western, eastern, central, and southern regions, respectively. All carcasses were submitted to the Veterinary Diagnostic Laboratory, Faculty of Veterinary Medicine, Khon Kaen University, for postmortem examination and further analyses.

### Sample collection and necropsy

The submitted birds were from flocks exhibiting acute onset of clinical signs, including lethargy, depre-ssion, increased mortality, and decreased productivity. The mortality rates among affected flocks ranged from 2.5% to 30%. Following necropsy, liver tissues were aseptically collected and immediately stored at −80°C pending molecular analyses.

### Histopathological examination

Liver samples were fixed in 10% neutral buffered formalin, routinely processed, embedded in paraffin wax, and sectioned. Tissue sections were stained with hematoxylin and eosin for microscopic evaluation of histopathological changes.

### DNA extraction and PCR amplification

Genomic DNA was individually extracted from liver tissues using the RBC real genomics viral nucleic acid extraction kit (Lot No. P3430-22047; RBC Bioscience, Taiwan) according to the manufacturer’s protocol. Amplification of a 590 bp fragment of the hexon gene was conducted using a previously described primer set by Marek *et al*. [26]: Hex L1-F (5′-ATGGGAGCSACCTAYTTCGACAT-3′, positions 301–323) and Hex L1-R (5′-AAATTGTCCCKRAANCCGATGTA-3′, positions 890–868) [27].

PCR was performed in a C1000 Touch Thermal Cycler (Bio-Rad, CA, USA) using DreamTaq Green PCR master mix (2×) (Lot No. 2873664; Thermo Fisher Scientific, MA, USA). Each 25 μL reaction mixture contained 3 μL of DNA template, 12.5 μL master mix, 0.5 μL of each primer (10 μM), and 8.5 μL nuclease-free water. Thermal cycling conditions included an initial denaturation at 95°C for 10 min; 40 cycles of denaturation at 95°C for 15 s, annealing at 56°C for 30 s, and extension at 72°C for 45 s followed by a final extension at 72°C for 5 min.

Positive and negative controls were included in all reactions. A previously confirmed FAdV-positive sample served as the positive control, while nuclease-free water was used as a substitute for the DNA template in the negative control. Amplified products were resolved through electrophoresis on 1.2% agarose gels stained with RedSafe Nucleic Acid Staining Solution (JH Science, WA, USA). The gels were visualized under ultraviolet illumination using a Gel Doc™ XR+ system and Image Lab™ software (Bio-Rad).

### Sequencing and phylogenetic analysis

Six PCR-positive samples, each from a different farm and province, were selected for DNA sequencing. Sequencing reactions were performed using the BigDye Terminator v3.1 cycle sequencing kit (Thermo Fisher Scientific), followed by purification and analysis using the Applied Biosystems 3730XL DNA Analyzer (Bionics, Seoul, South Korea).

Nucleotide sequences were identified through basic local alignment search tool (BLAST) analysis (version 2.16.0; https://blast.ncbi.nlm.nih.gov/Blast.cgi). Low-quality bases were trimmed using the Appl-ication Binary Interface chromatograms in the BioEdit software package (version 7.7.1, https://bioedit.software.informer.com/7.7/). Multiple sequence align-ments of study isolates with reference sequences from GenBank were performed using ClustalW in MEGA version 11 [28]. Genetic distances were computed using the Kimura 2-parameter model, and phylogenetic trees were constructed through the neighbor-joining method with 1,000 bootstrap replicates. Accession numbers for the six Thai FAdV isolates (PQ588151–PQ588156) and reference strains are provided accordingly.

## RESULTS

### Gross pathology

**Figure 1:**
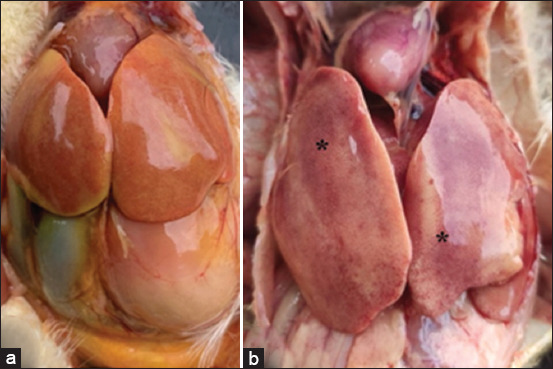
Macroscopic appearance of chickens with FAdV infections in Thailand. (a) A 3-day-old broiler breeder infected with FAdV-E serotype 8b presented the livers were markedly enlarged, friable, pale yellow, and multiple petechial hemorrhages. (b) Thirteen-day-old broiler infected with FAdV-E serotype 8b frequently exhibited a distinctly enlarged liver with an accentuated lobular pattern (asterisk). FAdV=Fowl aviadenovirus.

In the majority of cases, the liver appeared markedly enlarged, friable, and pale yellow, with numerous petechial hemorrhages (Figure 1). Samples collected from 28-day-old broilers and 350-day-old broiler breeder chickens with reported increases in daily mortality exhibited mildly hemorrhagic hepatic lesi-ons. Detailed information on flock age, clinical signs, morbidity, and mortality is summarized in Table 1.

**Table 1:** Origin, age of affected chickens, percentage of morbidity and mortality, and main clinical signs.

No.	Province	Chicken age (days)	Number of birds in the flocks	Percentage of morbidity and mortality	Main clinical signs

Broiler farm					
1	Songkhla	4	35,000	8	Lethargy, increased daily mortality
2	Chonburi	5	10,000	10	Lethargy, increased daily mortality
3	Songkhla	28	35,000	4–5	Increase daily mortality, retardation
4	Lopburi	13	15,000	15	Lethargy, convulsion
5	Kanchanaburi	27	25,000	30	Lethargy, high mortality
6	Chonburi	18	16,500	6	Lethargy, high mortality

**Broiler breeder farm**					

7	Chonburi	210	6,000	2	-
8	Chonburi	350	6,500	2.50	-
9	Chonburi	6	9,000	10	Lethargy, increased daily mortality
10	Kanchanaburi	5	6,000	5	Lethargy, high mortality
11	Chonburi	3	10,000	5	Lethargy, high mortality

### Histopathological findings

**Figure 2:**
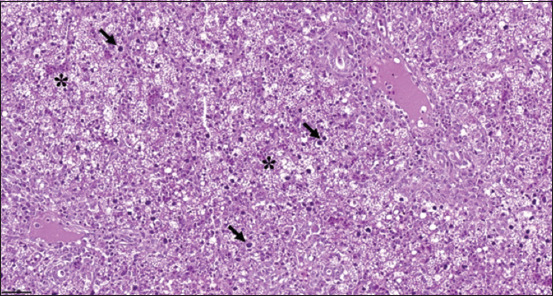
Microscopic finding of chickens with FAdV infections in Thailand. Liver: marked multifocal to coalescent necrosis of hepatocytes (asterisk) and hepatocytes containing noticeably basophilic intranuclear inclusion bodies (arrow). Hematoxylin and eosin. Obj. 40. Scale bar: 20 μm. FAdV=Fowl aviadenovirus.

Microscopically, liver tissue demonstrated multifocal to coalescent vacuolar degeneration and nec-rosis. Hepatocyte nuclei were diffusely enlarged and contained large, round basophilic intranuclear inclusion bodies, consistent with FAdV infection (Figure 2).

### Molecular detection of FAdV

**Figure 3:**
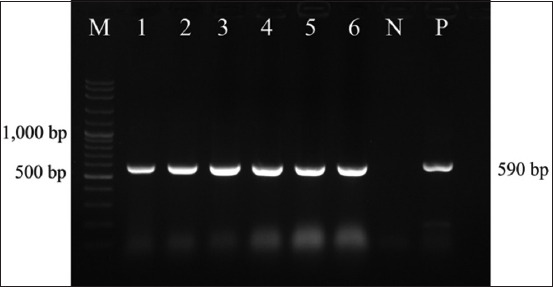
Amplification of hexon gene fragments of FAdV-E serotype 8b determinants on agarose gel approximately 590 bp. Lane M: 100-bp DNA ladder. Lane 1-6: Test samples. Lane N: Negative control. Lane P: Positive control. FAdV=Fowl aviadenovirus.

PCR amplification of liver DNA yielded specific 590 bp products from all 28 chicken carcasses, as confirmed by agarose gel electrophoresis (Figure 3). The PCR-positive samples were subjected to nucleotide sequencing for further characterization. Six representative isolates from the four surveyed provinces were selected for sequencing and submitted to GenBank under the following accession numbers:

- FAdV/KCB/KKU01-24 (PQ588151)
- FAdV/CB1/KKU02-24 (PQ588152)
- FAdV/CB2/KKU03-24 (PQ588153)
- FAdV/LB/KKU04-24 (PQ588154)
- FAdV/SK1/KKU05-24 (PQ588155)
- FAdV/SK2/KKU06-24 (PQ588156).

BLAST analysis confirmed the high sequence similarity of these isolates to the FAdV hexon gene.

### Phylogenetic analysis

**Figure 4:**
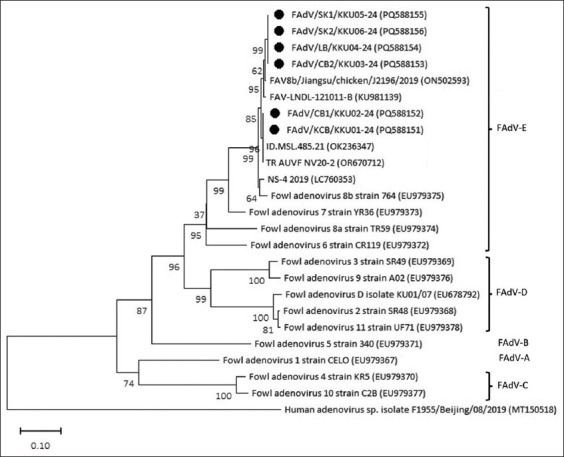
Phylogenetic tree of FAdV isolated from positive samples in Thailand. The six isolates in this study were marked with •whereas other isolates were available at GenBank with the accession number attached. The phylogenetic tree was constructed using the neighbor-joining method with 1000 bootstrap replicates on MEGA 11. The sequence of human adenovirus spp. F1955/Beijing/08/2019 accession number MT150518 was co-analyzed as an outgroup. FAdV=Fowl aviadenovirus.

Multiple sequence alignment and phylogenetic reconstruction revealed that all six Thai isolates belonged to FAdV species E, serotype 8b, exhibiting 94.73%–100.00% sequence identity with reference FAdV-8b strains from GenBank (Figure 4). Based on sequence clustering, the isolates were grouped into two distinct clades:

- Group 1 included four isolates (FAdV/CB2/KKU03-24, FAdV/LB/KKU04-24, FAdV/SK1/KKU05-24, FAdV/SK2/KKU06-24), which showed 97.82%–100.00% identity among themselves and 94.73%–99.02% similarity to reference strains.
- Group 2 comprised two isolates (FAdV/KCB/KKU01-24 and FAdV/CB1/KKU02-24), with 99.80%–99.81% similarity between them and 96.60%–100.00% similarity to reference isolates. Notably, FAdV/KCB/KKU01-24 demonstrated 100% nucleotide identity with strains from Turkey (OK236347) and Indonesia (OR670712).

### Amino acid comparison of hexon gene

**Figure 5:**
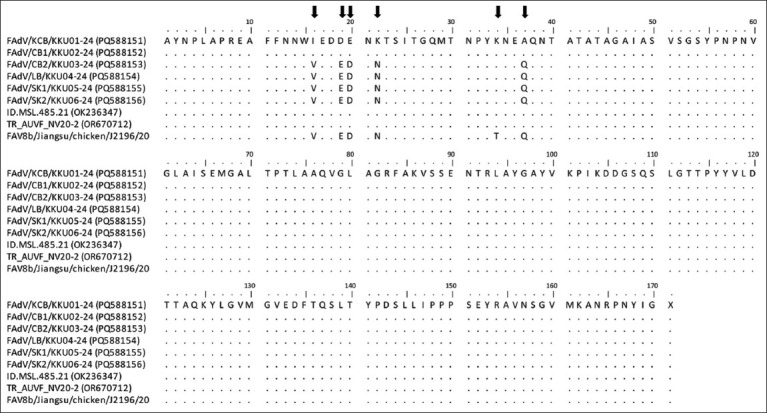
Amino acid alignments of the partial hexon protein of six FAdV-E serotype 8b isolated in Thailand. The black dots represent amino acids identical to those of FAdV/KCB/KKU01-24. Six amino acids are indicated by black arrows. FAdV=Fowl aviadenovirus.

Comparative amino acid sequence analysis of the partial hexon gene (Figure 5) identified five substitution sites distinguishing the two groups:

Positions 17, 19, 20, 22, and 37 differed between Group 1 and Group 2:

- Isoleucine → Valine
- Aspartic acid → Glutamic acid
- Glutamic acid → Aspartic acid
- Lysine → Asparagine
- Alanine → Glutamine.

Group 2 isolates (FAdV/KCB/KKU01-24 and FAdV/CB1/KKU02-24) shared 100% amino acid identity with the Turkish (TR_AUVF_NV20-2) and Indonesian (ID.MSL.485.21) isolates. In contrast, Group 1 isolates (FAdV/CB2/KKU03-24, FAdV/LB/KKU04-24, FAdV/SK1/KKU05-24, and FAdV/SK2/KKU06-24) differed from the Chinese reference strain at position 34, where lysine was substituted with threonine.

## DISCUSSION

### Global emergence and economic impact of IBH

IBH has been reported in broiler, broiler breeder, and layer flocks across various countries [12, 13, 25, 27, 29], with a notable rise in global incidence in recent years [30]. Affected poultry flocks commonly experience high mortality rates, reduced productivity, and compromised overall performance, culminating in substantial economic losses to the global poultry industry.

### Transmission dynamics: Horizontal and vertical routes

FAdV, the causative agent of IBH, is transmitted both horizontally through the fecal-oral route and vertically through embryonic infection [31]. In the present study, two broiler farms with confirmed FAdV infections had sourced their chicks from a common broiler breeder flock. This suggests the likelihood of vertical transmission, where infected breeder hens pass the virus to their progeny through embryonated eggs. FAdV transmission is known to occur during peak egg production due to stress-induced viral reactivation, with progeny typically becoming infected within 3–6 weeks post-hatching [32].

Vertical transmission thus appears to play a pivotal role in disseminating the virus across provinces. In addition, the observed genomic similarities among isolates from different geographic regions support potential epidemiological linkages and common origins of infection [33]. Horizontal transmission may fur-ther propagate the virus through feces, contaminated equipment, farm personnel, vehicles, and mechanical vectors such as flies, rodents, and wild birds [33].

### Serotype identification and geographic correlation

Our results confirm that recent IBH outbreaks in multiple Thai provinces were associated with FAdV-E serotype 8b. This serotype has also been identified in other regions, including Malaysia [34], South Korea [35], Poland [36], Turkey [27], South America [37], Brazil [29], and China [38], indicating its widespread global distribution and potential for cross-border dissemination.

### Implications for vaccination and biosecurity

Vaccination remains a key preventive strategy. Inactivated vaccines derived from FAdV serotypes 2 and 8b have demonstrated efficacy in inducing maternal immunity when administered to broiler breeders, thereby protecting young chicks [39, 40]. Recently, recombinant subunit vaccines, such as the Fiber-8a/8b-AD construct, have been developed as promising alternatives to conventional whole-virus vaccines [41]. In parallel, stringent biosecurity measures, including disinfection protocols and improved management practices, are essential to minimizing the risk of infec-tion [42].

### Diagnostic utility of histopathology

Histopathological assessment remains a cornerstone for the diagnosis of FAdV infections in poultry. The hallmark presence of basophilic intra-nuclear inclusion bodies in hepatocytes, observed in our study, corroborates findings from previous reports by Witoonsatian *et al*. [25] and Muhammad Redzuan *et al*. [34] on FAdV-D serotype 2 in Thailand and FAdV-E serotype 8b in Malaysia. This reinforces the diagnostic relevance of microscopic liver examination for FAdV confirmation [23].

### Phylogenetic grouping and genetic variability

Molecular characterization of the partial hexon gene in this study revealed two distinct phylogenetic clusters of FAdV-E serotype 8b. Notably, isolates within the second group, despite originating from different provinces, were traced back to the same parent bre-eder flock, supporting the hypothesis of vertical tran-smission. This finding highlights the importance of routine genetic surveillance to monitor the evolution and dissemination patterns of the virus.

Furthermore, our analysis revealed amino acid mutations in the hexon gene that align with findings from Malaysia, where 10 substitutions were identified in isolates collected between 2004 and 2011 [43]. Similar mutations in FAdV serotype 2 and serotype 8b have been implicated in heightened pathogenicity and mortality, particularly in IBH cases in specific-pathogen-free chickens [44, 45]. In Indonesia, circulating FAdV-8b strains between 2017 and 2019 exhibited analogous amino acid changes in the L1 loop region [9], suggesting ongoing genetic drift that may influence viral virulence and epidemiology.

### Limitations and future directions

Although this study provides valuable insights into the molecular epidemiology and genetic diversity of FAdV-8b in Thailand, the relatively small sample size may limit the generalizability of our findings. Future studies should incorporate broader geographic sampling and complete genome sequencing to comprehensively understand the evolutionary dynamics and pathogenic mechanisms of FAdV.

To mitigate future outbreaks, it is imperative that poultry farmers implement robust disease monitoring programs and track molecular changes in FAdV strains during epidemic events.

## CONCLUSION

This study represents the first comprehensive report of IBH outbreaks associated with FAdV serotype 8b (species E) in commercial broiler and broiler breeder farms across multiple provinces in Thailand. All 28 liver samples from affected chickens tested positive for FAdV, and phylogenetic analysis of the hexon gene revealed that the Thai isolates belonged to FAdV-8b, clustering into two genetically distinct groups. Amino acid comparison identified five key mutations differentiating these groups, with some isolates showing 100% identity to strains previously reported in Turkey and Indonesia. These findings suggest simultaneous circulation of multiple FAdV-8b lineages, likely spread through vertical transmission routes through breeder flocks.

The detection of distinct phylogenetic clusters among geographically dispersed farms underscores the importance of molecular surveillance in tracing viral transmission pathways. The histopathological evaluation confirmed the diagnostic reliability of baso-philic intranuclear inclusion bodies in hepatocytes as hallmarks of IBH. This integrative approach – combining gross pathology, histology, PCR-based detection, and molecular phylogenetics – enhanced diagnostic accuracy and epidemiological insight.

From a practical standpoint, the study underscores the urgent need for targeted vaccination programs that utilize locally circulating FAdV strains. Inactivated and subunit vaccines specific to FAdV-8b could sign-ificantly reduce mortality, improve productivity, and minimize economic losses in Thailand’s poultry sector. Furthermore, enhancing biosecurity measures and controlling vertical transmission from breeder farms should form a cornerstone of FAdV prevention strategies.

A major strength of this study lies in its mult-idisciplinary methodology, encompassing field investi-gation, molecular diagnostics, and comparative genomics, which collectively provide a comprehensive understanding of FAdV-8b epidemiology in Thailand. However, the study’s limitation is its modest sample size, which may restrict extrapolation to the national level.

In conclusion, this research contributes critical insights into the genetic diversity and transmission dynamics of FAdV-8b in Thai poultry populations. It provides a scientific foundation for the development of region-specific control measures and underscores the necessity for continuous genomic surveillance to preempt future IBH outbreaks.

## AUTHORS’ CONTRIBUTIONS

SJ and TP: Designed and conducted the study and drafted and revised the manuscript. SJ, DK, and KS: Performed the tests. KW: Supervised the study. All authors have read, reviewed, and approved the final manuscript.
